# Patients’ satisfaction with heroin-assisted treatment: a qualitative study

**DOI:** 10.1186/s12954-023-00808-8

**Published:** 2023-06-13

**Authors:** Rune Ellefsen, Linda Elise Couëssurel Wüsthoff, Espen Ajo Arnevik

**Affiliations:** 1 Section for Clinical Addiction Research, Oslo University Hospital, PO Box 4959 Nydalen, 0424 Oslo, Norway; 2 The Norwegian Centre for Addiction Research, University of Oslo, PO Box 1039 Blindern, 0315 Oslo, Norway

**Keywords:** Heroin-assisted treatment, Diacetylmorphine, Opioid use disorder, Patient satisfaction, Qualitative research, Socio-environmental factors

## Abstract

**Background:**

Heroin-assisted treatment (HAT) involves supervised dispensing of medical heroin (diacetylmorphine) for people with opioid use disorder. Clinical evidence has demonstrated the effectiveness of HAT, but little is known about the self-reported satisfaction among the patients who receive this treatment. This study presents the first empirical findings about the patients’ experiences of, and satisfaction with, HAT in the Norwegian context.

**Methods:**

Qualitative in-depth interviews with 26 patients in HAT were carried out one to two months after their enrollment. Analysis sought to identify the main benefits and challenges that the research participants experienced with this treatment. An inductive thematic analysis was conducted to identify the main areas of benefits and challenges. The benefits were weighed against the challenges in order to assess the participants’ overall level of treatment satisfaction.

**Results:**

Analysis identified three different areas of experienced benefits and three areas of challenges of being in this treatment. It outlines how the participants’ everyday lives are impacted by being in the treatment and how this, respectively, results from the treatment’s medical, relational, or configurational dimensions. We found an overall high level of treatment satisfaction among the participants. The identification of experienced challenges reveals factors that reduce satisfaction and thus may hinder treatment retention and positive treatment outcomes.

**Conclusions:**

The study demonstrates a novel approach to qualitatively investigate patients’ treatment satisfaction across different treatment dimensions. The findings have implications for clinical practice by pointing out key factors that inhibit and facilitate patients’ satisfaction with HAT. The identified importance of the socio-environmental factors and relational aspect of the treatment has further implications for the provision of opioid agonist treatment in general.

## Background

Heroin-assisted treatment (HAT) is an intensive form of treatment for opioid use disorder (OUD) that involves the dispensing of medical heroin (diacetylmorphine) from clinics where additional psychosocial interventions and support services are often available. Internationally, this medication is typically not available in a take-home form, unlike other medications used in opioid agonist treatment (OAT). Norwegian HAT patients are expected to be present in the clinic twice daily for supervised intake of medical heroin.

HAT is considered an evidence-based approach for a highly vulnerable patient group [1, 2]. Results from randomized controlled trials suggest that HAT can be effective in reducing crime and illicit heroin use [3] and that the target group stays in this treatment longer than in traditional OAT [4, 5].

A large body of research in the OUD field demonstrates that the risk of overdose is high before entering treatment and even greater when treatment is terminated [6]. Around 1 out of 10 OAT patients in Norway terminate their treatment annually [7], while a recent systematic review found that between 20 and 84% of OAT patients remain in treatment [8]. For people with OUD, it is particularly important to adjust and tailor treatment options to different needs in order to facilitate longer treatment retention and reduce serious harm [9]. HAT is therefore considered an important option for this particular group of patients.

Treatment satisfaction among patients who receive addiction treatment is considered an issue of significance in both clinical practice and research [10, 11]. Treatment satisfaction includes patient evaluation of their own experiences of receiving treatment and health-care services [12]. Patients who are satisfied with OAT tend to stay in treatment longer [9]. A survey including 1939 patients in outpatient treatment for substance use disorders (SUD) found that satisfaction was positively associated with either the completion of treatment or longer treatment retention, which is further related to favorable treatment outcomes [13].

Patients’ treatment satisfaction is not limited to the pharmacological aspect of treatment but is also influenced by socio-environmental factors associated with the clinical staff [14, 15]. These factors include the staff’s continuity, their personal beliefs about illicit drug use, their preferred methods of treatment, and their therapeutic skills [16, 17]. Patients’ satisfaction with the medication offered in OAT also positively influences their satisfaction with other interventions offered in the treatment centers [18]. Patient satisfaction with prescribed medical heroin cannot, therefore, be detached from the setting in which HAT is provided and the way clinicians provide it [19].

How clinicians relate to and provide services to patients is fundamental to the patients’ experience of the treatment [20]. In treatment with medical heroin, one study found that unfavorable interactions with providers of medical heroin or hydromorphone treatment had the strongest independent effect on how patients’ satisfaction changed over time [21]. Patients see relational dynamics, such as those related to their trust in the clinicians and the clinical environment, as issues that are significant predictors of satisfaction over time [22]. Given the significance of relational factors for patients’ overall perceptions of treatment, scholars have called for future studies to help determine the inhibitors and facilitators of positive patient–clinician relationships [21]. Our study answers this call by employing an innovative multidimensional approach where we distinguish between the three key dimensions that are crucial for patients’ satisfaction with HAT: the medical dimension (the diacetylmorphine), the relational dimension (the patient–clinician interactions), and the configurational dimension (the configuration of the treatment).

Only a limited number of studies have examined satisfaction among HAT patients. Moreover, the studies that exist are primarily based on quantitative methodology. Qualitative data enable greater insight into the social and relational aspects of treatment [23]. Qualitative studies have also demonstrated their ability to identify treatment outcomes that are often overlooked in clinical trials, like the treatment’s positive impact on self-esteem [24]. The successful effect of HAT thus suggests that the pharmacology of the drug is not the only key to a favorable treatment outcome [25]. This study therefore employs a multidimensional approach that covers the medical, relational, and configurational dimensions of HAT. This enables us to identify which dimension of HAT is associated with each benefit and challenge that the patients experience in this treatment.

## Methods

### Study context

Norway’s first HAT clinics opened in 2022 as a five-year trial project for people with OUD who have not benefited sufficiently from existing OAT. HAT exists in Canada and seven European countries, but the treatment is configured slightly differently across these countries [26]. Provision of medical heroin is the core component of this treatment, while other aspects, such as the number of doses provided per day, may differ.

Norwegian HAT is organized in the specialized health services as a part of the established system for OAT. However, HAT differs from traditional OAT by its different operating methods (e.g., higher intensity) and the use of medical heroin. The configuration of HAT in Norway means that patients may spend up to two hours at the clinic daily. This involves time spent in the waiting room, prescreening conversation, the injection room, and the mandatory observation (min. 20 min) for those who inject the medication. This reflects the intensity of this treatment and illustrates why the clinical environment and patient–clinician interactions are crucial for how this treatment is experienced.

Norwegian HAT patients are offered two heroin intakes daily, with the option of less frequent attendance. For the periods not covered by medical heroin, the patients were offered take-home methadone during this study. New national OAT guidelines introduced after this study also made slow-release oral morphine available for HAT patients to take home. At the time of this study, the majority of enrolled HAT patients administered the diacetylmorphine by injection, while about 5% used the alternative oral route of administration. In this National context, HAT is provided in two designated clinics as part of a trial project. The clinics are primarily staffed by nurses, social educators, social workers, and medical doctors with specialization in addiction medicine. Here, the patients are offered basic health checks in addition to the medications, while staff engage in milieu therapy and assist users on issues like finances and housing. These services may not be provided in all HAT clinics internationally [27].

HAT is open-ended and with no time-limit for patients in Norway, although continuation after the 5-year trial period is unsure. The psychosocial support and care offered are voluntary-based, and no engagement with additional services is required. The poly-drug use among the patients is acknowledged by clinicians and does not in itself lead to sanctions. However, an observational screening by the staff is carried out before medication is provided, and the dosage is reduced if a patient is too affected by other drugs or alcohol.

At the time of writing (June 2023), about 70 patients were enrolled in HAT in Norway’s two largest cities (Oslo and Bergen). Bergen’s clinic was using a temporary location at the time of this study, with a limited capacity of only 20 patients (the capacity increased to 40 in 2023). The clinic in Oslo has a physical capacity of between 70 and 90 patients, according to staff. The stated aim is to increase the total number to between 150 and 300 patients nationally within the period of the trial project [7]. It is still uncertain whether HAT will become a part of the established OAT treatment services and expanded after the trial period ends. Empirical studies involving primary data and results from Norwegian HAT is yet non-existent. The present study thus fills a knowledge gap by providing insights into the patients’ initial experiences and satisfaction with HAT in Norway.

### Participants, setting, and data collection

This study is based on interviews with 26 individuals (Oslo: *N* = 19, Bergen: *N* = 7) enrolled in HAT. These participants were between 31 and 68 years of age with 47 as the mean age and consisted of 20 males and 6 females. Two participants received heroin in tablet form while the rest injected it. Interviews were conducted four to eight weeks after the participants started HAT. The interviews took place inside or just outside the HAT clinics between March and July 2022. The users’ frequent visits to the HAT clinics made it possible to meet them repeatedly and build trust before conducting the interviews. Participants were not offered any compensation.

A six-person research team carried out the interviews, including two peer researchers with lived experience of OUD from the OAT user organization ProLAR Nett. The researchers had no previous relations to the recruited participants. Questions for the semi-structured interviews were designed to capture patients’ positive and negative experiences with HAT and its impact on their everyday lives. This included questions about what they were most satisfied with and what they found most challenging with HAT, what they thought could be different in HAT, as well as questions regarding the medications, relationship with clinicians and the treatment scheme. Interviews were conducted shortly after HAT was established in Norway, which means that the clinics were in a start-up phase with a number of patients that was lower than what was planned for during a normal situation in the clinics.

Interviews were recorded and lasted on average 41 min. They were conducted after medication intake to have a calmer setting for conversation. The researchers spent considerable time in the clinics to establish rapport before recruiting participants. We used the same opioid intoxication scoring tool as used in Norwegian HAT to make sure researchers never asked for consent or conducted interviews if patients were too affected by the medication. The tool is a translated version of the one used in Danish HAT clinics [28]. Gaining participants for the interviews was challenging in terms of being able to meet users at a time when they were both willing and not too affected by the medications to participate. Participants’ capacity and willingness to talk about the issues raised in interviews varied greatly. This is likely to be related to their varying capability of self-reflection and self-expression, their mental state on the day of the interview, and the related influence of medical heroin and other drugs [29]. To accommodate these challenges and the varying accessibility to the participants we employed a flexible approach. This involved considering the patients’ state and situation at the time of every scheduled interview, which led to numerous postponements and cancellations of interviews. For some patients, we facilitated their participation by dividing the interview into several shorter conversations.

All participant names below are pseudonyms. To protect anonymity, we also omitted information about the city in which participants had been enrolled and their age.

## Results

Transcribed interviews were coded and analyzed following the principles of a flexible inductive thematic analysis [30]. We started out identifying and distinguishing between the experienced benefits and challenges of being in HAT, including their positive and negative impacts on patients. The analytical process involved creating and revising codes (themes) to capture the most prevalent benefits and challenges, resulting in three elements of the treatment that were beneficial and three that were particularly challenging. The resulting structure of codes is used to report our findings, where we also link each benefit and challenge to the dimensions (the medical, relational or configurational) of the treatment that produced them (see Table 1).

**Table 1:** Patients’ experienced benefits and challenges of being in HAT

Benefits	Challenges
Access to medical heroin (medical)	Treatment scheme (configurational)
• Reduced stress and financial pressure	• Lack of medications
• New routines and hope	• Too intense
Patient–clinician relations (relational)	Clinic rules (configurational)
• Respectful engagements	• Unfounded rules
• Being heard	• Negative influence on relation with clinicians
Supporting environment (configurational)	Downtime and uncertainties (configurational and relational)
• Opportunities for psychosocial assistance	• Too much downtime
• Safer usage setting	• Uncertainties about project’s future

### Benefits of the treatment

Three aspects of HAT stand out as beneficial: the access to medical heroin, the positive patient–clinician relations, and the supportive environment of the clinic.

#### Access to medical heroin

This aspect stands out as the most crucial to the participants and is related to the medical dimension of the treatment. Access to medical heroin was beneficial in two ways: First, it helped to reduce the stress linked with constant pressure to acquire money for illicit drugs. Secondly, the daily clinic visits introduced new routines that—combined with medical heroin—provided energy and hope.

Ingrid compared her life before and after receiving medical heroin:

I would wrap drugs into portion packs, sell them on the streets, being all day around people that are stressed and who want drugs and do not have enough money […] And in the middle of it, maybe if you're lucky you'll sell some sex on the corner too, right, it's just… from that life to being able to sort of wake up, come here to get medicine and then go home, go to bed and sleep two hours on the couch, like. […] It brings a calm and a peace over my everyday life and my life which is completely… well, I've spent over €200 a day on drugs.

Entering treatment with medical heroin alleviated the constant financial pressure to raise money for heroin, as Anne explained: “Life has changed in the way that it has become more quiet at home.” She and her partner needed €600 a day to avoid withdrawal. Many participants sold drugs to finance their own use of illegal heroin before entering HAT. Karl used to sell amphetamines but explained how the pressure to sell changed once he “no longer needed as much money” to stay well. Martin and others similarly said “I don’t sell drugs like I used to, since I started here.”

Related positive impacts of medical heroin were outlined by Geir: “It simply enables me to use my brain capacity for something else than chasing heroin.” Reducing the stress of hustling money impacted the participant’s life, like Tor explained: “I love that I have stability now. That I know, every day, I don’t need to stress about it. That I have what I need. It means a lot, like, to my quality of life.”

The medical heroin created predictability regarding a need that had to be covered in the users’ daily life. These positive impacts are likely to have broader mental health benefits, as alleviating stress positively contributes to people’s recovery processes [31]. Many participants compared the stress of acquiring money for illegal heroin with the demand of meeting up frequently in the clinic. Fredrik said “Even if we have to come here two times a day, and it is kind of impractical and stuff, it’s peanuts,” because before entering treatment there were not “enough hours around the clock to stress about money.”

Reduced financial pressure further contributed to make the second positive impact of medical heroin possible: new routines and positive energy. Despite the intensity of the treatment, many users found the routines and structure of regular clinic visits positive. The behavioral change of new daily routines imposed by receiving medical heroin in HAT was described by Arild: “I just notice how much easier it is to get out now and getting things done at home, and I eat more.”

Turid pointed out a cognitive shift related to having more time: “Clearly, I have much more time to stake out the life and path I wish for. […] I feel that I can take a look around me and look for opportunities with my eyes wide open.”

HAT structured the lives of participants through the regular clinic visits. These visits and the regular medication intake impacted their everyday routines. Alex felt that entering treatment both offered routine and positive energy: “At last, I have something I can go to regularly. And now I notice that I am starting to get inspired again.”

Erik noticed similar life changes: “It has become a lot better.” He explained “Earlier I just woke up, right? Now I have something to go to.” Erik started to laugh and said that treatment was “almost like being at work again” as he got into a daily “rhythm.” Several participants referred to being in treatment as a job. Ingrid was one of them: “To come here, the rhythm of having something to fill my days with […] It’s like… I see it as my job [laughs] to come here every day. And it has become a very nice job.”

Turid initially “thought it would be a problem” with the intensity of the treatment, but the medical heroin made it “easier to go outside” because “my body feels lighter. It’s easier to just be, to exist.” Many patients referred to the benefits of medical heroin compared to methadone because medical heroin made them function better, both cognitively and physically.

Receiving medical heroin has been found to promote changes in patients’ outlook [1], while studies also find that regular supervised intake of medical heroin provides valuable stability and routines to patients’ lives [24].

#### Patient–clinician relations

The positive relations between patients and clinical staff were prevalent in the patients’ stories and refer to the relational dimension of the treatment. This dimension overlaps with what others have conceptualized as “the therapeutic relationship” [32] or “everyday interactions” between users and providers of treatment services [33]. Participants described two aspects of the positive patient–clinician relations: the respectful interactions with staff, and the experience of having an influence on their own treatment.

Referring to previous treatment experiences, Anne described her entry into HAT: “Wherever you go really, you are used to being met with raised eyebrows or a kind of skepticism. And I just have to praise the people working here. I like them all.” Many patients voiced that the staff treated them better than the way they were used to, and often contrasted this to earlier experiences in traditional OAT. Ann described HAT as “the exact opposite” and continued: “I struggled with anxiety, struggled to get out and meet people. And that’s not how it works here at all. Like, it’s like I am a totally different person here.” The positive relationship with clinicians mattered.

Thomas put into words a difference between traditional OAT and HAT: “I think it’s kind of… it’s a better culture here, I think. I think we are met with more respect, and that they have a different approach to us as drug users” Making similar comparisons, Erik said: “It’s like night and day!” and explained the difference: “You get treated for who you are, and it’s not the rules and regime that you have in OAT.”

Geir was positively surprised by HAT: “People are treated respectfully, and kind of get… they aren’t kicked around, and then they behave a lot better. There is a better unison, really.” Fredrik was most satisfied with “those who work here, and kind of the whole atmosphere, the whole way of being welcomed.”

These positive relations with clinicians represented something unusual to several participants. Ingrid said “one simply isn’t used to being met with openness and trust and humanity.” Similarly, Stian found HAT to be unlike his former experiences:

I feel they have knowledge. Perhaps not every person of the staff. Some are new to learning about it, but they behave professionally towards us. And those who are in charge, in particular, have great understanding for the issues, and they adjust the treatment to us.

The reciprocal trust and respect between staff and patients stand out as a key feature of what makes their interaction a positive experience for participants. Stian further felt this treatment was tailored to patients, and not the other way around. This brings us to the second positive aspect of patient–clinician interactions: patients having their voices heard and having influence on their own treatment.

Stian continued to explain: “You can tell them what you have taken. As long as it does not go against what you are about to take [medical heroin], you get the dose you are supposed to get, and you have influence on the dosage.” He described his experience of both increasing and decreasing the dosage: “My voice gets heard—it’s user participation, as it is so nicely called.”

Erika experienced having a great impact on her own treatment: “For example, we adjust my dosage upwards when I need a bigger dose.” Ingrid shared the same experience: “Yes, they listen to what I say, right?”.

Adjusting and finding the right dosage was a major issue among the participants. Influence on dosages was mentioned as a clear sign of being heard and taken seriously by staff. Ola described how the medical doctor had “been absolutely fantastic” in following up on him and in “finding a dose that is adapted to me, that makes me able to feel well and good.”

The positive patient–clinician relations are likely to contribute to a strengthened feeling of self-worth, as opposed to the stigma that patients experienced in other settings. Experience of stigmatization in treatment for OUD is generally a major barrier to treatment entry and retention [34]. The positive experiences of interacting regularly with the clinicians added important meaning to the clinic visits. Positive relations are generally important for the recovery processes of persons in OAT [35].

#### The supportive environment

This beneficial aspect of the treatment was pointed out by many participants and is related to the configurational dimension of the treatment. The configurational dimension covers the way in which the treatment is organized and configured; for example, the services that are provided and that may differ among clinics. Two attributes of the supportive environment were emphasized as beneficial: First, the variety of psychosocial and medical assistance offered and secondly, the way the clinic and treatment provided for a new structure around—and safer setting for—the patients’ heroin use.

When asked if he would recommend HAT to others, Erik answered “Absolutely!” He continued explaining why: “Help is provided here, you know, and they are here for you.” Most participants gave examples of support and assistance they had received from staff. Fredrik said “Firstly, they helped me sort out my finances. So I am working on that now actually.” Thomas offered another example:

When I started here, I got appliances in place in my flat, firstly. […] When I took my medication just now, the nurse said to me: “Have you been to Jysk [warehouse selling beds and bedding products] yet?” And I just said, “No. That’s true… I have a voucher for Jysk.” I will try going there later today to use it.

Fredrik expressed that he was “almost allergic to social workers” but said that what the social worker in HAT had been able to get in place for him was “brilliant in every way.” He received help applying for economic support from the social service, and described assistance with health appointments and a hospital visit. Entering HAT seemed to lower the threshold for using services for many participants, by making patients more able and willing to follow up on different social and health issues.

Anne told us: “I have already been called in to a check with the heart specialist because of a strange sound in the heart. That’s something the medical doctor started right away. […] And then there is that job project.”

Marius was satisfied with the help he received: “I think I have received good help from the social worker, ’cause I am actually in the middle of a housing crisis.”

Fredrik described benefits of being assisted in health issues:

They figured out that I had a very low level of vitamin D, so now I get a vitamin D supplement here, every day. And I have talked to the social worker…. With his help I have booked an appointment at the dentist's and things like that.

Elin also had received “lots of” help and emphasized the impact it had on her: “I am shocked. I have gotten a hope I didn’t have before.” The supportive environment triggered Elin’s hope.

Participants did not follow up or follow through on all the opportunities and assistance that the clinic provided or offered. Nevertheless, many described that they had initiated and followed up more on issues that were important to their everyday lives, health, and quality of life. Birger eagerly told us: “Now I am starting to work tomorrow” through help from the staff. He continued: “I have never worked in my whole life.”

Several participants described the second benefit of the supportive environment; feeling cared for and being safer in HAT than they were outside of treatment. Erika said “At least we have a social worker, a psychologist, and we have six nice nurses and good medical doctors… we now have a good team around us.” Ola had years of experiences from the health-care system before entering HAT: “I have had all kinds of diseases, and I have never been in a unit or anything where I have felt this much at home and welcomed and so well taken care of.”

If a patient in HAT has an overdose or is too heavily affected by medical heroin intake, clinicians are prepared for instant medical aid. Patients also inject in a clinical setting with clean syringes and medical-grade heroin, which does not cause the same problems as illicit street heroin, including less abscesses and other health risks. Vein scanners are available to help users find suitable veins for injection, while clinicians also offer guidance for injecting in the large muscles as an alternative to the veins.

A feeling of being safer and taken care of was important for the participants’ perception of the treatment (see also [36]). Reidar underlined his hope for care in HAT: “It’s the medical things. That they can follow up on my health, my kidneys, and that they are able to help me, like getting me off the methadone.” Martin said this about HAT: “It has helped, not mainly about the money, but firstly about my own life. I don’t get overdoses. I don’t get abscesses. I don’t get this and that. It’s safety.”

The patients’ experiences of HAT as a supportive environment are closely related to the positive patient–clinician relationships and the previously described benefits of medical heroin. The latter enables patients to use the supportive environment, while the former makes the clinic visits something positive.

The intake of medical heroin overseen by health personnel offered a secure setting with assistance and care that marked a radical shift in the participants’ heroin use patterns prior to HAT [see [19] for similar findings]. The barriers for participants to make use of services and assistance also seemed to be lowered by entering HAT. This increased use of psychosocial support is likely to positively impact the patients’ quality of life and treatment outcomes [11].

### Challenges of the treatment

Three aspects of HAT stand out as challenging to the participants: the treatment scheme, the clinic rules, as well as the increased downtime and uncertainties about HAT’s future.

#### Treatment scheme

This aspect stands out as the most important challenge for the participants. It is related to the configurational dimension of the treatment, and particularly the way in which the treatment and medication provision is organized. Patients described two main challenges: First, the limited types, level, and frequency of medications available in HAT; and secondly, the inconveniences of having to show up at the clinic twice a day.

Related to the former, what Ingrid found most challenging was: “Perhaps that it’s only open two times a day [laughs]. It should have been a third time just before the night. It’s hard to get the medication to cover myself around the clock.” The opening hours of the Norwegian HAT clinics are limited to the periods of about 8 am–12 pm and 2–5 pm. Most patients received take-home methadone to cover the evenings and nights, but many disliked its negative side effects or lack of desirable effect. As a result, many chose to buy illegal heroin and other drugs to cover this period. Ingrid hardly used methadone at all and wanted to replace it with slow-release morphine, if possible. Erika said “I think there should be a broader choice of medications. There ought to be a lot more types of morphine. I would have liked to get a morphine tablet.”

Complaints about methadone were widespread (see also [36]). Fredrik did not like it: “Methadone, it’s like… it makes you well, but oh my god you get so parked in the head that it’s mad. In that way, that’s one of the few things that I see as somewhat negative here.” Marius thought some things in HAT should be changed:

The most important change, I think, would be to open up for other drugs as well. Amphetamines and… yeah. Like, I am here because I have a problematic relationship with heroin, and if the goal is to abstain fully from drug crimes or that type of lifestyle, then those [amphetamines] ought to be offered here as well. At least acknowledge that people are using them, and that it should be allowed to use them here.

Similarly, Thomas explained “I am also dependent on amphetamines” and he wished that HAT could offer amphetamines to those dependent on them.

The experience of receiving too little heroin or take-home medication for the evening and night led many participants to buy illegal drugs. Thomas explained “Here they say: ‘It’s supposed to be enough.’ No, it’s not, because I get fucking sick.” Thomas and many others addressed this challenge of getting by during the nights.

Reidar described such an incident: “What the hell, I get very sick and have a lot of pain and such when I am being stepped down [receiving reduced dosages]. It makes me have to buy heroin.” Many patients used illegal drugs in addition to the legal medication they received, but this was clearly influenced by what the clinic offered.

Some participants used illicit heroin preemptively to avoid waking up sick, because the medical heroin they received was usually not enough to avoid symptoms of withdrawal. Others described being able to skip illegal heroin during the night, but this was dependent on getting an appropriate afternoon heroin dosage in the clinic.

The question of dosage and how to get enough and suitable medication to avoid withdrawal were key issues that featured across interviews. There is no set maximum dosage of medical heroin in Norwegian HAT. Several participants wished to obtain a higher heroin dosage. Anne was not satisfied: “I think the adjustments of my dose are somewhat slow.” She still expressed understanding for the reason behind it, which was her irregular attendance in the clinic.

Anne and a few others had problems with getting to the clinic during opening hours, which relates to the second experienced challenge of the treatment scheme: its intensity. The intensity of the treatment scheme was described as challenging because frequent visits within limited opening hours reduced their opportunity for other activities. They also spent much time inside the clinics in addition to traveling back and forth for each visit. Kjetil found it challenging that HAT “takes quite some time.”

When asked if the treatment made it easier to follow up on other things he wanted to do, Ola replied: “Yeeah [hesitant]. It’s both ways, really, but it’s the thing that you have to show up here two times – you must keep that in the back of your mind all the time. You need to adjust the rest of your life around that.” Like many other participants, Ola underlined that the frequent visits “might make it harder to plan other things.” Martin described the mixed feeling about the treatment’s intensity:

I come every day, twice a day, twice a day. I must like it, or otherwise, what the fuck? I don’t like to wake up, I don’t like to be here at 8 am every day. I come every day, but it’s hard.

Erika wanted a part of the treatment scheme to be different: “The thing about picking up twice daily; it could be allowed to get heroin to take on a vacation and out of here, but that’s not possible.” Stian also described challenges of going on vacation. He was prepared to go on vacation with the methadone he would be given to take with him, but: “I have sorted out some Oxycodone illegally, like, to use as a supplement.” These and other individual challenges, particularly for those who disliked methadone, featured in several interviews. Their solution was usually to buy illegal heroin and other drugs.

The patients’ use of illegal drugs while in HAT should be seen in relation to the configuration of the treatment. This includes the types, frequency, and levels of medication offered, and whether it makes patients feel covered from symptoms of withdrawal. Several participants discussed going with relatives on holiday trips but found it challenging or impossible because of the limited alternatives of take-home medications that could replace medical heroin. The intensity of the treatment could also create extraordinary challenges for patients who had physical disabilities or traveling distances of more than one hour to the clinic. These burdens of the treatment scheme have also been emphasized as negative by HAT patients in other studies [21].

#### Clinic rules

Clinic rules were described as challenging by many participants. This is related to the configurational dimension of the treatment, and specifically the way in which patients’ medication intake and behavior are regulated during their clinic visits. Clinic rules were challenging in two ways: They were experienced as too strict or unfounded, and the clinicians’ enforcement of rules and sanctions negatively influenced their relations with patients.

The clinic rules most often referred to by participants were those regulating behavior in different sections of the clinic; in the waiting, screening, injection, and observation rooms. In the injection room, these rules set a limit of 20 min with three attempts to inject the medication. Rules also cover the visual-based screening of patients before heroin intake and a minimum of 20 min of mandatory observation after injection. The visual screening before intake involves the potential for a reduced dose, or denial of medical heroin, if patients are too affected by other drugs or alcohol. Such dosage reduction was at times perceived as an unreasonable sanction or even punishment.

Turid gave an example: “There’s a few others who have gotten their doses reduced because they have been quite loaded when they arrived here, and they experienced it as a sanction.” She had received a reduced dose herself after she told clinicians that she had taken some pills:

Not that I am going around lying to them, but I will not tell them the next time if I have a slip on pills or something else. They will have to figure that out themselves. I may get punished for it if I say something. […] They reduce your dose and in addition they demand that you are supposed to get by and stay well.

The experienced sanctions influenced the relationship with clinicians. Håvard also experienced having his dosage reduced: “Yes, if I have been too drugged, yes, I have. When you are too loaded you are not served.” He found these experiences hard to talk about, but Håvard and other participants usually expressed understanding for having their dose reduced.

Fredrik, however, described a rule that many experienced as unfounded:

What I find somewhat strange is the rule that you are not allowed to shoot in the groin, and those things. I cannot understand…. I get the feeling that this is a rule that is created for you [providers of treatment] more than us. […] I think people should be able to shoot where they used to. It’s not a problem in the injection room [a supervised drug consumption site], so why should it be a problem here?

Marius had negative experiences of the rules related to late arrival as well as the limited time and injection attempts in the injection room:

If you arrive one minute too late, you are not let in. And you have 20 minutes to inject. And they are quite meticulous about it… to begin with, it was three attempts. But now it has dropped to two, but they describe it as three. Like, there’s quite a few things I experience as somewhat strict. […] To me, it has become a big issue to actually make it on time, simply.

Some participants raised the negative implications of having the ban and the 20-min limit in the injection room, which caused stress for patients in a way that could lead to bad decisions and unsafe injection practices.

There had been incidents in the clinic where patients covertly injected in the groin, despite the ban. The ban was a big issue for participants who had been injecting in the groin for years because they had few or no alternative veins to use. After being caught injecting in the groin, Thomas was called to a “serious talk” with the staff about the rules and what was expected of him. Repeatedly failing to abide by the rules could lead to suspension from HAT for a limited time period. Serious violations of rules, like violent behavior or severe threats of violence, may lead to immediate discharge. No patients had been discharged on these grounds between HAT’s start in January 2022 and the time of writing (June 2023).

Martin found the mandatory 20-min observation period in the clinic after each injection to be troublesome:

Sometimes when we are done shooting, why do they tell us to sit down 20 minutes? You don’t need 20 minutes, I don’t understand. Maybe to check; that’s alright. Sometimes some people who come here get mad at them [staff], they scream and this and that. And sometimes when the time is over, they [staff] tell you, go! It’s finished – go!

What Martin describes here, and parts of what Marius said above, are not about the rules themselves, but the way in which they are enforced by clinicians. This brings us to the second challenge of clinic rules: Their enforcement sometimes negatively impacted patient–clinician relations.

Some patients reacted to the arguments used by staff to legitimate the clinic rules, as they were seemingly not primarily based on medical reasoning or considerations of the patients’ health. Patients expressed that some clinic rules could contribute to discontent among users, a worsened atmosphere in the clinic, and a tenser relationship with the clinicians.

Birger mentioned what he perceived as the most demanding part of the treatment rules: “It has to be the thing with waiting for the dose.” All patients have to remain in the waiting room together with other patients before they are allowed into the screening room prior to heroin intake. Stian mentioned wanting the opportunity “to report criticism” about clinic rules and how the clinic is run to the staff and leadership.

While many were clear that several clinic rules were frustrating and negative, a widespread theme was still that HAT was experienced as much better than traditional OAT, because the HAT rules were perceived as more laxed and involving less sanctions (see also [37, 38]). However, HAT still involves a set of rules and potential sanctions that regulates the patients’ behavior in the clinic. Other studies also find that patients have negative experiences with the enforcement of clinic rules in HAT [21].

#### Downtime and uncertainties

This challenge includes having too much downtime and uncertainties about the future of HAT. Concerning the latter, uncertainties about whether HAT will be terminated after the 5-year trial project ends is related to the configurational dimension of the treatment, while concerns for the clinic milieu with increasing numbers of patients is linked to the treatment’s relational dimension. The challenges of downtime are also related to the configurational dimension of the treatment, especially regarding which activities are offered and possible for patients in HAT.

While the benefits of having more time as a result of access to medical heroin were described above, this also involved challenges. Many participants voiced how downtime caused unrest or boredom. Fredrik was one of them: “With all the spare time I suddenly have gotten, there’s a bit of time to sit and ponder about life, and about everything that did not turn out the way it should.” Ingrid thought the hours between the two daily clinic visits was the worst period: “It’s kinda like I wonder a bit about what more we are to do eventually, yeah. And I know there are more people that are kind of calling for something more.”

When Thomas was asked if he felt less or more socially isolated after entering HAT, he explained:

Suddenly I am sitting there you know with my flat, empty apartment, and myself and I don’t know what the fuck to do, you know. And it becomes… it’s been kind of empty […] I get kind of scared by it. It’s kind of gloomy, right? So I hope they make some initiative down there [in HAT], someone should have done that here, you know, taken some initiative for people to being able to… ’cause there are loads of us here who want to do things now.

Thomas explained another implication of having too much downtime: “I know several people here are still just going downtown, you know, pushing, or going there because they don’t have anything else to do maybe. I don’t fucking know, like. I am doing the same thing myself.”

Karl echoes several participants when saying: “I had expected it to be something more, like, than just coming here for a shot, kind of.” He continued: “It would have been good to have something to do during the days. It’s of course possible to continue as before, going downtown pushing drugs… that’s an option too.” Obviously, getting more free time does not automatically influence positively on recovery processes [38].

Both Karl and other participants had opportunities for assistance in HAT for job training, courses they might be interested in, or going to a psychologist, while the social workers and other staff also assisted the patients in using opportunities for activities outside of HAT if they wished to. Be that as it may, many did not use these opportunities. Several participants missed social and cultural activities to fill their time between the first and second clinic visit, but what HAT offered did not seem to cover what they sought. Stian had therefore taken an initiative on his own: “We are trying to, a bit on our own, to set up some space for music rehearsal somewhere close to the clinic, so we can have something to do after the shot.” He said that this was missing “also for patients who don’t like music, but who just want to draw, or have some place to socialize.”

The second set of challenges include uncertainty about what the future for HAT will be with more patients and what will happen if HAT is terminated after the 5-year trial period. Anne felt well taken care of in HAT, but realized that the situation would change:

That’s why I am so chuffed that I am in on this in the start-up. Oh my god, all those people [clinicians], and they have so much time as well. Person number 50 is not going to have the same experience that I had as one of the first 15 persons.

Håvard voiced similar concerns: “The only thing I am thinking about is the number of people that will be coming here. That’s what I am thinking. It can be problematic.”

The concern over increasing numbers of patients is related to the atmosphere and milieu in the clinic. The reduced time of clinicians per patient was one issue raised, but participants also voiced concerns about patients queuing outside the clinic before the opening hours and felt that this might lead to quarrels about who is to enter first.

Stian was among the first people who started receiving medical heroin. He emphasized the solidarity and absence of thieving in the beginning, but that “it seems like there has been some lately.” The concern about more conflicts among patients as their number rises was voiced by several participants. Thomas was one of them: “There’s been a few troublemakers coming lately who have triggered the atmosphere in a bit of a negative direction.”

Ola mentioned a challenge that few others raised: “I don’t like the drug scene in this town. I have always tried to avoid it. That’s the only negative thing with HAT, that it’s a gathering of users in one place.” And thinking about the future, he said “When there’s more participants, users, in this system, it means we are going to be gathered and meet every day and it’s gonna involve, most likely, people you don’t want to meet.” Similar concerns also featured among HAT patients in previous studies [21].

The challenging uncertainties in HAT involved concerns that the atmosphere among users, the positive experience of being in the clinic, and the good relations with clinicians could deteriorate. Concerns were also raised about whether HAT will continue after the 5-year trial period, and how it will affect them if medical heroin becomes unavailable. Martin raised this issue: “It’s a good place, but how long will it last? I don’t know.”

Thomas related the uncertain future of HAT to his experience from another treatment trial project for SUD:

I was part of RusFact [Flexible Assertive Community Treatment for people with SUD] that was just down the road here, but it was a trial project that was terminated, like suddenly. And it’s an example of… I have been through so many processes with so many people which are like… I open up, I enter it with full energy, show up, and I have really fucking done it, showing up. Even with my disability I went there every fucking day, showing up at what I was supposed to show up for, and those kinds of things. Then, suddenly it’s just: ‘Well, now we are dissolving this, because it was only a trial project.’ So all the relations you made there, now it’s just – fuck you, kind of.

The issue of concerns over HAT’s future was not among the most prominent challenges in our data. However, research from other HAT trials has shown that patients have experienced the exit from such trial projects as “tumultuous,” and that they were “anxious about their future” as the trial neared its end and they were to be transitioned to treatments that had failed them in the past [27].

## Discussion

This qualitative study from the Norwegian context has outlined the three most prevalent benefits and challenges of being in HAT, as seen from the patients’ perspective. Access to medical heroin, the positive patient–clinician relations, and the supportive environment of the clinic and wider treatment were experienced as main benefits. The most challenging aspects were the intense treatment scheme and its limitation in the medications provided, the strict clinic rules, as well as the increased downtime and concerns over the lastingness of HAT. Assessing patients’ treatment satisfaction in this study thus involves weighing their experienced benefits against the challenges.

It is evident that participants were more satisfied than dissatisfied with entering and being in treatment, one to two months after their enrollment. From what participants described as changes in their everyday life after entering treatment, it is also clear that their quality of life has improved in certain areas. Being in HAT helped make their everyday lives safer, more predictable, stable, and with less constant pressures to commit crimes or obtain money in undesirable ways (for similar findings, see [3, 39–43]) These benefits are likely to contribute positively to treatment retention. The fact that many patients experienced a welcoming environment in HAT, new positive routines, and valuable relations with clinicians as well as gratitude regarding the supportive treatment environment seems—in sum—to give participants a greater sense of self-determination. This is also related to their experience of being heard and having an influence on their own treatment, which enhances patients’ “relational autonomy” [42]. These positive treatment outcomes are likely to assist patients’ recovery processes by contributing to the user’s “positive sense of identity apart from one’s condition while rebuilding a life despite or within the limitations imposed by that condition” [43]. People with OUD, either in or out of treatment, view the social life and the daily activities as most important for their recovery processes [44]. The sum of benefits seemingly makes many patients experience stronger personal relationships, which involves social inclusion and increased self-determination in their everyday life: benefits that have been emphasized as fundamental to the quality of life for people with OUD [45].

Each benefit and each challenge were related to different dimensions of the treatment (see Table 1). The medical dimension of the treatment—primarily the stable access to medical heroin—covered patients’ opioid dependency, but had key social, financial, and mental health implications, which often contributed positively to participants’ quality of life. The relational dimension of the treatment involved regular positive interactions with staff that constituted a meaningful activity in itself, a counterweight to the stigma that patients experienced in other settings (see also [46–48]). The relational dimension of the treatment was most often described as challenging in relation to staff’s enforcement of clinic rules and when staff members were perceived as lacking in knowledge about heroin use and injection practices. The benefits identified with the relational dimension of the treatment and the positive patient–clinician relationships were closely related to the experienced absence of hostility and intrusive controls. Most of these patients had strong, negative experiences from earlier treatment in traditional OAT.

While HAT patients are likely to have a longer series of negative experiences with OAT than the average OAT patient, and Norwegian OAT has changed substantially over the years, the difference in atmosphere and relationships still stand out as a core contrast between HAT and traditional OAT. HAT’s configuration seemingly enables closer relationships in a milieu where patients and clinicians get to known each other by both formal and informal modes of daily interactions. This is usually not the case in traditional OAT.

Despite restrictive regulations of HAT which in principle is similar to, and in some ways more restrictive than traditional OAT, the patients were still much more satisfied with HAT. The medication was one crucial difference, but the experience of how the staff were flexible and respective toward patients and their expressed needs stand out as equally important (see also [49–51]). The clinicians seemed to exercise flexibility in a way that alleviated some of the challenges of the restrictive treatment setting.

Most of the experienced challenges were in relation to the configurational dimension of the treatment, which involves the way it is organized and provided to patients. The treatment scheme’s intensity and the limited range, level, and frequency of medications offered were the most prevalent challenges featuring in the data. This duality in the patients’ experience reflects a general tension between the patients’ need for and satisfaction with structured care in HAT, and the restrictive setting in which it is provided, that patients did not like.

This study covers the short-term impacts of the treatment, where the patients’ satisfaction concerns the transition into treatment and the experience at the onset of HAT. Satisfaction may of course change over time, but at this point, the benefits of being in HAT outweighed the challenges involved. Some areas of the treatment are still pointed out as being particularly challenging. The participants’ lives are bound up in a highly intense treatment scheme which may become more challenging if the benefits dissolve over time: for example, if the clinic environment and interaction with staff become negative experiences. The results of this study should thus be interpreted in relation to the phase of the treatment that data covered as well as the fact that HAT was newly established and not yet in normal operation with the full number of patients.

Explaining the different dimensions of the treatment that produce the specific benefits and challenges that patients experience, contributes by indicating trajectories and mechanisms through which specific elements of the treatment produce certain outcomes. This helps fill a gap regarding why and how HAT produces these outcomes. While the access to medical heroin seems to produce similar positive outcomes for patients in HAT across countries, such as reducing illicit heroin use and contributing to safer injection practices [50], the relational dimension of the treatment is likely to differ more across clinics and countries as it depends more on contextual factors like the organizational culture of the clinic as well as the type of staff and their views of OUD patients [51].

These findings may be used to create more user-oriented services. Assessing the impact of clinic rules and whether their intentions are achieved seem particularly important in HAT across contexts. Whether opening hours or the number of medication doses offered daily should be expanded, or if groin injections should be allowed in combination with guidance and follow-ups are issues that could be considered in the Norwegian context. In Germany and Switzerland, HAT clinics offer up to three and five medication doses daily, wider opening hours and supervised groin injections [52]. Switzerland also has positive experiences with take-home medical heroin to stable patients [53]. Such user-oriented configurations of HAT may make it more flexible toward patients’ needs. However, take-home medical heroin may weaken the positive impact of the therapeutic relationship between patients and clinicians, which is based on the frequent clinic visits.

Knowledge generated from this study may inform current and future HAT programs in Norway and beyond. As this study covers a phase of HAT with a high level of satisfaction, it may be used as a point of comparison in later studies of patients’ satisfaction with HAT domestically and as a template for similar qualitative studies abroad. The insights about what is experienced as positive and negative across each treatment dimension could be useful for OAT in general, where the insights about what enables the positive patient–clinician relations seem particularly important.

## Conclusions

This study employed a novel multidimensional approach to investigate patients’ self-reported satisfaction across the medical, relational, and configurational dimensions of HAT. It is clear from data that the benefits clearly outweigh the challenges when the patients’ experienced benefits with HAT are weighed against their experienced challenges. We thus found a high level of treatment satisfaction among patients. The findings have implications for clinical practice by pointing out key areas of the treatment that should be maintained—or potentially changed—to ensure a high level of satisfaction over time. The identified importance of socio-environmental factors and the relational dimension of the treatment also has broader implications for the provision of OUD treatment services more generally. It provides insight into key factors that make the patient–clinician relationship something positive, meaningful and therapeutic in itself.

## Data Availability

The interview transcripts generated during the interviews in this study are not publicly available to preserve the confidentiality of the participants.

## Abbreviations

HAT: Heroin-assisted treatment
OAT: Opioid agonist treatment
OUD: Opioid use disorder
SUD: Substance use disorder
